# Evolving Systemic Therapy for Prostate Cancer: Pivotal Clinical Trials, Biomarker-Driven Combinations, and Practical Sequencing in the ARSI–PARP–Radioligand Era

**DOI:** 10.3390/cancers18121966

**Published:** 2026-06-17

**Authors:** Takatoshi Somoto, Takanobu Utsumi, Rino Ikeda, Tatsuharu Sugimoto, Naoki Ishitsuka, Yodai Kadono, Takahide Noro, Yuta Suzuki, Shota Iijima, Yuka Sugizaki, Ryo Oka, Takumi Endo, Naoto Kamiya, Hiroyoshi Suzuki

**Affiliations:** 1 Department of Urology, Toho University Sakura Medical Center, Sakura 285-8741, Japan; takatoshi.soumoto@med.toho-u.ac.jp (T.S.); rino.ikeda@med.toho-u.ac.jp (R.I.); naoki.ishitsuka@med.toho-u.ac.jp (N.I.); takahide.noro@med.toho-u.ac.jp (T.N.); yuta.suzuki@med.toho-u.ac.jp (Y.S.); shouta.iijima@med.toho-u.ac.jp (S.I.); yuuka.kizuki@med.toho-u.ac.jp (Y.S.); ryou.oka@med.toho-u.ac.jp (R.O.); takumi.endou@med.toho-u.ac.jp (T.E.); naoto.kamiya@med.toho-u.ac.jp (N.K.); hiroyoshi.suzuki@med.toho-u.ac.jp (H.S.); 2 Department of Urology, Toho University Graduate School of Medicine, Ota-ku, Tokyo 143-8540, Japan; tatsuharu.sugimoto@med.toho-u.ac.jp (T.S.); yodai.kadono@med.toho-u.ac.jp (Y.K.); 3 Department of Urology, Seirei Sakura Citizen Hospital, Sakura 285-8765, Japan; 4 Department of Urology, Mihama Hospital, Mihama-ku, Chiba 261-0013, Japan

**Keywords:** metastatic prostate cancer, treatment sequencing, androgen receptor signaling inhibitors, PARP inhibitors, PSMA-targeted radioligand therapy

## Abstract

Systemic therapy for metastatic prostate cancer is increasingly defined by upfront intensification and biomarker-guided mechanism switching. This narrative review synthesizes pivotal randomized trials, guideline recommendations, and implementation-focused literature across metastatic castration-sensitive prostate cancer (mCSPC) and metastatic castration-resistant prostate cancer (mCRPC). Evidence was organized around decision points at mCSPC diagnosis and at mCRPC transition, while incorporating biological mechanisms of resistance, including AR-axis reactivation, AR splice variants, lineage plasticity, DNA repair–hormone signaling interactions, and PSMA expression heterogeneity. In mCSPC, androgen deprivation therapy plus docetaxel and/or androgen receptor signaling inhibitors (ARSIs) improves survival, with triplet regimens favored for selected chemotherapy-fit patients with aggressive de novo disease. In mCRPC, cross-resistance limits routine ARSI-to-ARSI switching, and randomized data support mechanism-distinct options, including taxanes, PARP-based therapy in homologous recombination repair (HRR)-altered disease, and PSMA-targeted radioligand therapy (RLT) in selected PSMA-positive patients. As RLT moves earlier, PSMA heterogeneity, renal function, bone marrow reserve, and emerging dosimetry-based optimization should inform practical implementation. Ongoing trials are evaluating earlier theranostics, alpha-emitting radioligands, and biomarker-enriched combinations. An implementation-first approach that intensifies treatment when appropriate, tests early and acts on HRR results, uses PSMA PET to guide RLT, and preserves hematologic reserve may maximize access to multiple life-prolonging mechanisms.

## Simple Summary

Treatment for metastatic prostate cancer has changed rapidly. Many patients now benefit from earlier combination treatment rather than hormone therapy alone. This review explains how major trials have reshaped care in metastatic hormone-sensitive and castration-resistant disease. We summarize who may benefit from upfront intensification, why DNA repair testing should be arranged early, and how PSMA PET can identify candidates for targeted radioligand therapy. We also explain why resistance to one androgen receptor pathway inhibitor may limit benefit from another, including AR reactivation, lineage plasticity, DNA repair vulnerability, and heterogeneous PSMA expression. The goal is to help clinical teams choose mechanism-distinct treatments while preserving hematologic reserve and adapting to comorbidity, access, and monitoring capacity.

## 1. Introduction

Systemic therapy for metastatic prostate cancer has evolved from a linear strategy of introducing one new agent at a time to a deliberately staged pathway that begins at diagnosis and anticipates the constraints of later treatment lines. The 2025 European Association of Urology–European Association of Nuclear Medicine–European Society for Radiotherapy and Oncology–European Society of Urogenital Radiology–International Society of Urological Pathology–International Society of Geriatric Oncology guideline update reflects this shift by positioning treatment intensification as the default strategy for most men with metastatic castration-sensitive prostate cancer (mCSPC) and by incorporating imaging- and biomarker-informed choices into algorithms for metastatic castration-resistant prostate cancer (mCRPC) [1]. In parallel, Posdzich et al. characterized the current era as one in which the optimal initial decision cannot be separated from later sequencing, because cross-resistance and cumulative toxicity may restrict downstream options when early treatment steps are not planned deliberately [2].

Two implementation realities now accompany efficacy. First, germline and somatic deoxyribonucleic acid (DNA) damage repair alterations have become actionable in routine care, but their actionability depends on when and how testing is delivered. A practice-oriented workflow for genetic testing and counseling across urologic oncology, including metastatic prostate cancer, emphasized that testing is a longitudinal care process rather than a single laboratory event and that it must be integrated with consent, referral pathways, and family-based cascade considerations [3]. Second, metastatic burden itself has become a moving target because PSMA PET has improved detection and reclassified many patients previously considered to have low-volume disease; this shift affects both trial interpretation and real-world selection of intensification strategies [4]. Finally, inherited DNA repair mutations are sufficiently prevalent in metastatic prostate cancer to justify early germline assessment, as shown in a landmark multicenter study, supporting the view that testing should be planned when metastatic disease is recognized rather than deferred until late mCRPC, when tissue and time may be limited [5].

This review links pivotal randomized trials to the points at which clinical decisions are actually made: selecting doublet versus triplet intensification at mCSPC diagnosis, determining when to change mechanism rather than switch within class in mCRPC, integrating homologous recombination repair (HRR) status into first-line mCRPC treatment selection, and using PSMA imaging to position radioligand therapy (RLT) without compromising eligibility for subsequent options. The emphasis is on practical clinical implementation and sequencing principles that treating teams can apply now.

## 2. mCSPC

The management of mCSPC has been transformed by evidence that earlier use of active agents improves survival compared with androgen deprivation therapy (ADT) alone. The practical question is no longer whether treatment should be intensified, but which intensification strategy is most deliverable and most likely to provide the greatest absolute benefit for an individual patient.

Chemohormonal intensification with docetaxel was established by CHAARTED, which showed improved overall survival with six cycles of docetaxel added to ADT in men with metastatic hormone-sensitive prostate cancer [6]. STAMPEDE confirmed the benefit of docetaxel added to long-term hormone therapy in a large platform-trial population, supporting external validity across routine practice settings [7]. In clinical practice, docetaxel is particularly compelling when rapid cytoreduction is required and the patient is fit for chemotherapy, especially in high-burden symptomatic disease. The trade-off is front-loaded toxicity. Febrile neutropenia, neuropathy, and functional decline can be decisive in older adults or in men with borderline performance status; these toxicities also matter because they may diminish the marrow and functional reserve needed for later poly(ADP-ribose) polymerase (PARP)-based therapy or RLT.

Chemotherapy-free intensification with ARSIs has broadened eligibility for early intensification. LATITUDE showed that abiraterone plus prednisone added to ADT improved outcomes in high-risk de novo metastatic disease [8]. ENZAMET and TITAN extended this evidence base to androgen receptor antagonists, demonstrating benefit with enzalutamide or apalutamide added to standard first-line therapy across broad mCSPC populations [9,10]. From an implementation perspective, ARSI selection is often shaped by comorbidity and monitoring capacity. Abiraterone requires concomitant corticosteroid use and careful monitoring of blood pressure, potassium, and hepatic function [8]. Androgen receptor antagonists may be limited by fatigue, falls, and cognitive vulnerability in selected patients [9,10]. The practical point is that ARSI toxicities are generally chronic rather than acute; successful intensification therefore depends on monitoring that can be sustained over years.

Triplet therapy has emerged as the most intensive evidence-based upfront strategy for selected patients. ARASENS demonstrated an overall survival benefit with darolutamide added to ADT plus docetaxel compared with placebo plus ADT plus docetaxel, with a manageable incremental toxicity profile [11]. PEACE-1 showed meaningful benefit when abiraterone was added to ADT plus docetaxel in de novo mCSPC, reinforcing that the incremental gain from maximal upfront systemic control can be clinically important in aggressive presentations [12]. In practice, triplet therapy is most appropriate for men who present de novo with high-volume or high-risk features and who are fit for chemotherapy. The decision should be explicit: triplet therapy is a deliberate choice to accept short-term chemotherapy-related toxicity in exchange for a higher probability of durable disease control and delayed need for subsequent therapeutic mechanisms.

The mCSPC landscape continues to evolve with respect to calibration and adoption. Kwon et al. emphasized that real-world uptake of doublet and triplet strategies remains heterogeneous and is strongly influenced by patient fitness, access, and clinician experience; this heterogeneity may itself widen outcome gaps across institutions [13]. De-escalation has therefore emerged as an active area of investigation, particularly for low-burden disease in the PSMA PET era, in which overtreatment is plausible and metastasis-directed or local approaches may be integrated in selected settings [14]. An updated network meta-analysis synthesizes first-line triplet and doublet therapies, supporting improved disease-control endpoints with triplets while underscoring the unavoidable limitations of indirect comparisons across trials with different populations, endpoint definitions, and follow-up durations [15]. For clinicians, these syntheses support a practical calibration principle: when chemotherapy is feasible and disease biology is aggressive, triplet therapy is appropriate; when chemotherapy threatens deliverability, ARSI-based intensification preserves benefit while reducing acute harm.

Darolutamide has also expanded into chemotherapy-free mCSPC. ARANOTE evaluated darolutamide plus ADT without docetaxel, supporting an additional chemotherapy-free intensification option for men in whom chemotherapy is undesirable or impractical [16]. This is operationally important because the decision to intensify is often constrained by logistics and comorbidity rather than by efficacy alone [16]. Table 1 summarizes pivotal and practice-shaping evidence in mCSPC.

## 3. Biological Basis of Treatment Resistance and Sequencing Decisions

The clinical rationale for mechanism switching in metastatic prostate cancer is grounded in the biology of treatment resistance. Although abiraterone and androgen receptor signaling inhibitors such as enzalutamide, apalutamide, and darolutamide differ in their direct pharmacologic targets, they ultimately converge on suppression of androgen receptor (AR)-dependent transcriptional activity. Therefore, resistance to one AR pathway inhibitor may reduce the likelihood of a durable response to another agent within the same functional class.

### 3.1. AR-Axis Reactivation and ARSI Cross-Resistance

Multiple mechanisms can restore AR signaling under therapeutic pressure, including AR amplification, AR overexpression, ligand-binding domain alterations, intratumoral androgen synthesis, altered co-regulator activity, and ligand-independent AR splice variants. Among these, AR-V7 is clinically relevant because it lacks the ligand-binding domain targeted by several AR-directed therapies and has been associated with resistance to abiraterone and enzalutamide in men with mCRPC [17,18]. In addition, preclinical models of resistance to abiraterone and enzalutamide show restoration of AR full-length expression, AR target-gene activity, and cross-resistance to the alternate novel hormonal agent, supporting a biological explanation for the limited value of routine ARSI-to-ARSI switching after progression [19]. These observations do not mean that every patient will fail sequential AR pathway inhibition, but they do justify prioritizing mechanism-distinct therapies when patients are fit and eligible.

### 3.2. Lineage Plasticity and Treatment-Emergent Neuroendocrine Prostate Cancer

AR-independent resistance represents a second and clinically important escape route. Under sustained AR pathway inhibition, some tumors acquire lineage plasticity and may evolve toward treatment-emergent neuroendocrine prostate cancer or other AR-indifferent phenotypes. This process is often associated with aggressive clinical behavior, discordance between PSA kinetics and radiographic progression, and reduced dependence on AR signaling [20]. Alterations involving TP53, RB1, PTEN, MYCN, AURKA, and epigenetic regulators have been implicated in this transition, although the precise mechanisms vary across tumors. From a sequencing perspective, suspected lineage plasticity should prompt reconsideration of continued AR pathway targeting and may favor taxane chemotherapy, platinum-containing approaches in selected cases, or clinical trial enrollment rather than another ARSI substitution.

### 3.3. DNA Damage Repair Biology and PARP Sensitivity

PARP inhibitor sensitivity is most clearly established in tumors with homologous recombination repair defects, particularly BRCA1/2 alterations. However, the magnitude of benefit differs across HRR genes, and not all HRR alterations confer equivalent PARP sensitivity. This gene-context dependence is important when interpreting PARP monotherapy and PARP–ARSI combination trials. Clinically, HRR testing should therefore be implemented early, but the therapeutic interpretation should remain gene-specific rather than treating all HRR alterations as biologically interchangeable.

### 3.4. Biological Rationale for PARP–ARSI Combinations

The rationale for combining AR pathway inhibition with PARP inhibition extends beyond simple co-administration of two active drugs. AR signaling regulates transcriptional programs involved in DNA repair, and second-generation antiandrogen therapy can downregulate DNA repair genes in prostate cancer models [21]. Conversely, PARP-1 has functions in both DNA damage repair and AR transcriptional regulation, supporting a mechanistic interaction between DNA repair biology and hormone signaling [22]. These observations provide a biological basis for PARP–ARSI combinations. At the same time, they also explain why clinical benefit may vary between HRR-altered and HRR-unselected populations and why hematologic toxicity must be weighed carefully when these combinations are moved earlier in the treatment sequence.

### 3.5. PSMA Expression Heterogeneity and Radioligand Resistance

PSMA-targeted radioligand therapy depends on sufficient and sufficiently homogeneous PSMA expression. PSMA PET can identify patients who are likely to receive target-directed radiation, but PSMA expression is not static and may vary between lesions. PSMA-low, PSMA-negative, or biologically discordant disease may be less suitable for PSMA-targeted treatment and should prompt consideration of alternative mechanisms, including taxanes or clinical trials. Thus, PSMA imaging should be interpreted not merely as a staging modality but as a biological selection tool within a broader sequencing strategy [4], as discussed in more detail in the PSMA-targeted RLT section below.

## 4. mCRPC

mCRPC is the phase in which the biological principles described above become clinically decisive. The key question is whether a patient can receive multiple mechanism-distinct life-prolonging therapies or will instead cycle through low-yield within-class substitutions. Because AR pathway reactivation, AR splice variants, and lineage plasticity can limit the effectiveness of sequential AR pathway inhibition [18,19,20], the first actionable principle is to avoid reflexive ARSI-to-ARSI switching after progression on one ARSI when a mechanism-distinct treatment is feasible.

CARD provided randomized confirmation that switching within class can be inferior to changing mechanism: in men previously treated with docetaxel and one androgen receptor-targeted agent, cabazitaxel improved outcomes compared with switching to the alternative androgen receptor-targeted therapy [23]. In the contemporary era, CARD is not merely a second-line trial; it is a sequencing anchor that supports early consideration of taxanes, PARP-based strategies in HRR-altered disease, and PSMA-targeted RLT when patients are eligible.

Taxane chemotherapy remains a durable therapeutic backbone, and its value is increasingly determined by timing and sequencing rather than by antitumor activity alone. A recent review that focused on taxane timing and sequencing argues that a chemotherapy-forward strategy is appropriate in selected contexts, while also clarifying how the availability of PARP combinations and RLT alters the therapeutic landscape and may justify earlier or later taxane positioning depending on treatment goals and eligibility [24]. This framework is clinically useful because it shifts the discussion from rigid line-based rules to scenario-based planning: the next best mechanism is selected not only for its activity but also for what it preserves.

The second actionable principle is to make the pathway biomarker-ready. HRR testing is now a care process. This framework provides counseling checklists and workflow elements for integrating germline testing and downstream actions into routine urologic oncology care, emphasizing universal testing considerations in metastatic prostate cancer and the need to connect results to treatment selection and familial implications [3]. The prevalence of inherited DNA repair gene mutations in metastatic prostate cancer provides an epidemiologic rationale for this approach and supports early germline assessment within an appropriate counseling infrastructure [5]. Operationally, early testing prevents delays at the transition to mCRPC, when tissue may be unavailable and progression may be too rapid to accommodate prolonged turnaround times.

PARP inhibitors progressed from concept to standard through a coherent sequence of trials. TOPARP-A established proof of concept that DNA repair-defective mCRPC may be vulnerable to PARP inhibition [25]. PROfound then demonstrated improved outcomes with olaparib compared with the physician’s choice of abiraterone or enzalutamide in HRR-altered mCRPC after ARSI progression [26], and survival analyses provided additional context in the setting of crossover and subsequent therapies [27]. TRITON3 expanded phase III evidence for PARP inhibition by supporting rucaparib over physician’s choice comparators in HRR-altered mCRPC [28]. Collectively, these trials support a practical principle: for HRR-positive disease, particularly that involving BRCA1 or BRCA2, PARP therapy should be planned as an intentional step rather than reserved as late salvage treatment.

The field is now increasingly shaped by PARP-ARSI combinations. PROpel showed improved radiographic progression-free survival with olaparib plus abiraterone versus abiraterone alone in an all-comer first-line mCRPC population [29], and final prespecified overall survival analyses further informed clinical interpretation [30]. MAGNITUDE used a biomarker-stratified design; the HRR-negative cohort was discontinued for futility, supporting benefit concentrated in HRR-altered disease and strengthening the case for biomarker-enriched use in many settings [31], with the final overall survival analysis adding important nuance [32]. TALAPRO-2 demonstrated robust disease-control endpoints for talazoparib plus enzalutamide in first-line mCRPC [33], and subsequent overall survival reporting further supports clinical relevance while leaving optimal patient selection open [34]. Clinical implementation depends heavily on toxicity planning. Anemia and fatigue are common, and myelosuppression may be amplified by prior chemotherapy, extensive bone disease, and baseline cytopenias. In this setting, sequencing becomes a form of hematologic toxicity management and preservation of bone marrow reserve: the order and timing of therapies associated with clinically relevant hematologic toxicity, including taxanes, PARP combinations, and RLT, can determine whether a patient remains eligible for the most effective later-line options.

The biological rationale for PARP–ARSI combinations is supported by bidirectional crosstalk between AR signaling and DNA repair. AR signaling can regulate DNA repair gene expression, whereas AR pathway inhibition may create a DNA repair-vulnerable state in selected prostate cancer models [21]. PARP-1 also supports AR transcriptional function in addition to its canonical role in DNA damage repair, providing a mechanistic explanation for why PARP inhibition may interact with AR pathway blockade [22]. However, these mechanisms should not be interpreted as evidence that all patients derive equal benefit from PARP–ARSI combinations. The clinical value of these regimens remains shaped by HRR gene context, prior therapy, baseline cytopenia, disease burden, and access.

Mechanistic and translational guidance can help clinicians understand why trial results differ. Rao et al. summarized the biological rationale and development considerations for androgen receptor-PARP co-inhibition, including why benefit may not be uniform across HRR genes and why biomarker-enriched strategies remain biologically coherent even when all-comer trials show some benefit [35]. Marchetti et al. situated PARP inhibitors alongside radiometabolic and theranostic approaches and emphasized that biomarker readiness and marrow reserve are central determinants of sequencing rather than secondary considerations, particularly as RLT moves earlier in the disease course [36]. Carr et al. reported a phase II trial of olaparib plus abiraterone with liquid-biopsy HRR characterization, providing additional context for how circulating tumor DNA (ctDNA) may support implementation and patient selection when tissue is limited [37].

Beyond HRR, other biomarker-enriched strategies are emerging. IPATential150 demonstrated improved outcomes with ipatasertib added to abiraterone in phosphatase and tensin homolog-loss disease, illustrating how selection beyond HRR may broaden the precision-medicine landscape [38]. Immunotherapy remains selective in prostate cancer; KEYNOTE-199 showed activity of pembrolizumab in a subset of patients, reinforcing that broad unselected use is not supported and that biomarker enrichment and trial design are central to identifying responders [39]. The taxane-focused review also discusses the realistic role of immunotherapy within modern sequencing, which may be useful when patients ask why checkpoint blockade is not a routine option in mCRPC [24]. Table 2 summarizes pivotal and practice-shaping evidence in mCRPC.

**Figure 1:**
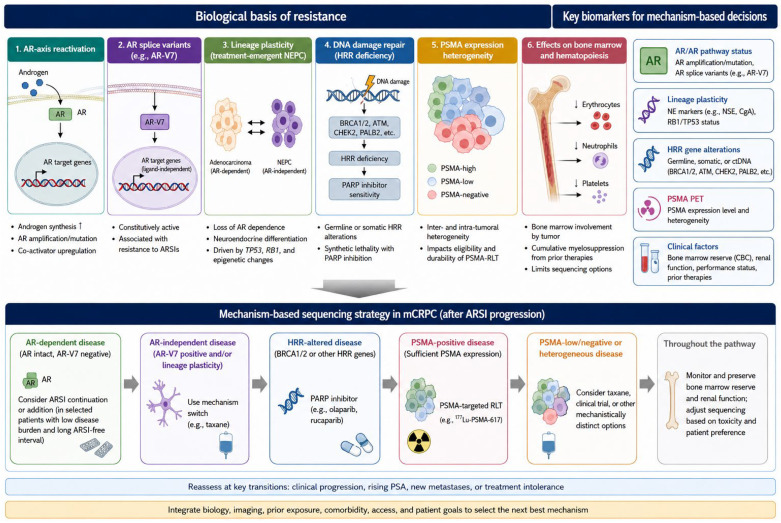
Biological basis of resistance and mechanism-based sequencing in mCRPC. The figure summarizes key mechanisms that influence treatment resistance and sequencing in metastatic castration-resistant prostate cancer, including AR-axis reactivation, AR splice variants, lineage plasticity, HRR deficiency, PSMA expression heterogeneity, and effects on bone marrow. The downward arrows beside erythrocytes, neutrophils, and platelets indicate potential decreases in these hematopoietic cell lineages, reflecting impaired bone marrow reserve or treatment-related hematologic toxicity. Biomarker and clinical assessment—including AR pathway status, HRR gene alterations, PSMA PET findings, bone marrow reserve, renal function, performance status, and prior therapies—can guide selection of mechanism-distinct treatments after ARSI progression. Treatment options include AR pathway-directed approaches in selected AR-dependent disease, PARP inhibitor-based strategies for HRR-altered disease, PSMA-targeted radioligand therapy for PSMA-positive disease, and taxane chemotherapy, clinical trials, or other mechanistically distinct options for AR-independent or PSMA-low/heterogeneous disease. Abbreviations: AR, androgen receptor; ARSI, androgen receptor signaling inhibitor; AR-V7, androgen receptor splice variant 7; ATM, ataxia telangiectasia mutated; BRCA1/2, breast cancer gene 1/2; CBC, complete blood count; CgA, chromogranin A; CHEK2, checkpoint kinase 2; ctDNA, circulating tumor DNA; DNA, deoxyribonucleic acid; HRR, homologous recombination repair; mCRPC, metastatic castration-resistant prostate cancer; NE, neuroendocrine; NEPC, neuroendocrine prostate cancer; NSE, neuron-specific enolase; PALB2, partner and localizer of BRCA2; PARP, poly(ADP-ribose) polymerase; PSA, prostate-specific antigen; PSMA, prostate-specific membrane antigen; PET, positron emission tomography; RB1, retinoblastoma 1; RLT, radioligand therapy; TP53, tumor protein p53.

Taken together, current mCRPC evidence supports a mechanism-switching strategy in which taxanes, PARP-based therapy, and PSMA-targeted RLT are prioritized according to biomarker status, imaging eligibility, prior exposure, and hematologic reserve (Figure 1).

## 5. Positioning PSMA-Targeted Radioligand Therapy Within Systemic Treatment Sequencing

In this systemic therapy-focused review, PSMA PET is discussed primarily as a treatment-selection tool rather than as a comprehensive imaging or theranostic platform. PSMA PET has changed the interpretation of disease burden and provides the imaging basis for selecting candidates for PSMA-targeted RLT, but the present discussion is limited to its relevance for systemic treatment sequencing [45,46,47].

VISION established lutetium-177 PSMA-617 (177Lu-PSMA-617) as a life-prolonging treatment for patients with PSMA-positive mCRPC previously treated with ARSIs and taxane chemotherapy [40]. TheraP further supported the antitumor activity of 177Lu-PSMA-617 compared with cabazitaxel in an imaging-selected population, although differences in eligibility criteria and trial design should be considered when applying these data to routine practice [41,42].

The most clinically relevant development for sequencing is PSMAfore. In taxane-naive PSMA-positive mCRPC after progression on one ARSI, 177Lu-PSMA-617 improved disease-control outcomes compared with changing to another ARSI, supporting RLT as a mechanism switch rather than another within-class androgen receptor pathway strategy [43,44]. At the same time, final overall survival in the intention-to-treat population was not statistically significant, a result that should be interpreted in the context of substantial crossover from the ARSI-change arm to 177Lu-PSMA-617. This finding extends the broader principle established by CARD: after key androgen receptor pathway exposures, changing mechanism is often more rational than simply changing brand within the same therapeutic class.

In practice, PSMA-targeted RLT should be positioned according to target suitability, prior treatment exposure, disease tempo, symptoms, marrow reserve, renal function, and local access to nuclear medicine infrastructure. Practice-oriented reviews and Japanese phase II data support the feasibility of translating RLT into real-world care, but patient selection remains critical [48,49]. For the purposes of this review, the central point is not the technical implementation of PSMA theranostics, but how PSMA-targeted RLT should be incorporated into a pragmatic ARSI–PARP–taxane–RLT sequencing framework.

Dosimetry and personalized theranostic optimization are becoming increasingly relevant as PSMA-targeted RLT moves earlier in the treatment sequence. Fixed-activity administration is practical and has enabled broad clinical implementation, but it does not fully account for interpatient variability in tumor burden, renal function, salivary-gland exposure, marrow reserve, and whole-body clearance. Organ and tumor dosimetry studies, including simplified protocols for [177Lu]Lu-PSMA-I&T, suggest that individualized dose estimation may help balance tumor absorbed dose against kidney, salivary-gland, and bone marrow toxicity [50]. At present, the main implementation barriers are additional imaging time points, institutional workflow, standardization of quantitative SPECT/CT methods, and uncertainty regarding how dosimetry should prospectively alter treatment decisions. Therefore, dosimetry should be discussed as a developing optimization tool rather than as a universally mandated requirement.

## 6. Practical Sequencing: An Implementable Pathway

**Figure 2:**
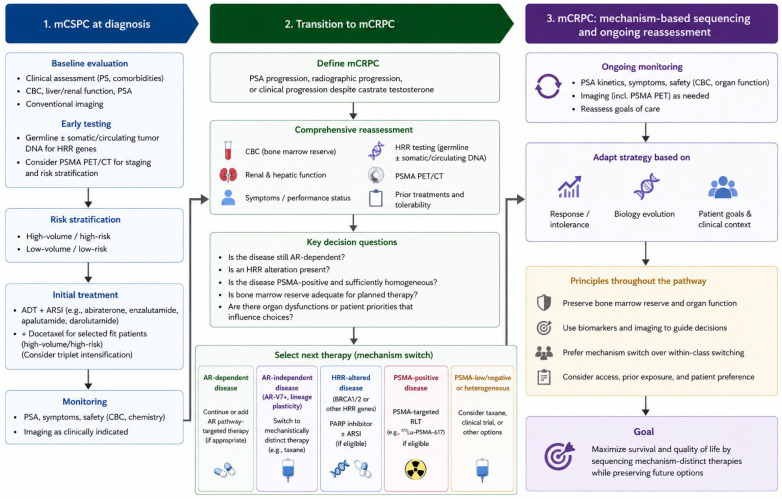
Implementation-first treatment algorithm from mCSPC to mCRPC. This algorithm summarizes practical treatment sequencing from mCSPC diagnosis to mCRPC transition and subsequent reassessment. At mCSPC diagnosis, treatment selection is guided by baseline evaluation, early HRR testing, imaging, disease volume/risk, and chemotherapy fitness. At mCRPC transition, comprehensive reassessment of disease progression, prior treatment exposure, HRR status, PSMA PET/CT findings, organ function, and bone marrow reserve supports mechanism-based treatment selection. In mCRPC, subsequent therapy should prioritize mechanism switching according to AR dependence, HRR alterations, PSMA expression, treatment tolerance, access, patient goals, and preservation of future treatment options. Abbreviations: ADT, androgen deprivation therapy; AR, androgen receptor; ARSI, androgen receptor signaling inhibitor; AR-V7, androgen receptor splice variant 7; BRCA1/2, breast cancer gene 1/2; CBC, complete blood count; CT, computed tomography; ctDNA, circulating tumor DNA; DNA, deoxyribonucleic acid; HRR, homologous recombination repair; mCRPC, metastatic castration-resistant prostate cancer; mCSPC, metastatic castration-sensitive prostate cancer; PARP, poly(ADP-ribose) polymerase; PET, positron emission tomography; PS, performance status; PSA, prostate-specific antigen; PSMA, prostate-specific membrane antigen; RLT, radioligand therapy.

The current evidence can be translated into an implementation-first treatment algorithm that begins at mCSPC diagnosis and anticipates biomarker-guided and imaging-guided decisions at mCRPC transition (Figure 2).

The available evidence does not mandate a single universal sequence, because regulatory approvals and access differ across regions and patient fitness varies widely. It does, however, support sequencing principles that can be implemented in routine care (Table 3).

Sequencing begins at the diagnosis of mCSPC. For chemotherapy-fit patients presenting with de novo high-volume or otherwise aggressive disease, triplet therapy is reasonable and often preferred because it maximizes early disease control and is supported by randomized evidence [11,12]. For patients in whom the risks associated with chemotherapy threaten deliverability, ARSI-based intensification often provides the best balance between efficacy and safety [8,9,10,16]. The key question is not whether docetaxel is active, but whether the short-term toxicity is justified by the incremental benefit compared with an ARSI-only strategy for that individual. The mCSPC decision should be made with downstream eligibility in mind, particularly preservation of marrow reserve for future PARP combinations or RLT.

The pathway should be biomarker-ready before mCRPC develops. Germline testing and counseling workflows provide a practical template for integrating testing into metastatic care and linking results to both treatment decisions and familial implications [3]. The prevalence of inherited DNA repair gene mutations in metastatic prostate cancer supports early implementation of this workflow [5]. Somatic profiling and ctDNA-based approaches may complement tissue testing when biopsy material is limited; phase II data incorporating liquid-biopsy HRR characterization illustrate how this approach may be operationalized [37]. The practical objective is to avoid a situation in which a patient develops mCRPC and progresses rapidly while the treating team is still attempting to obtain tissue or arrange counseling.

At the transition to mCRPC, therapy selection should be guided by the dominant actionable axis and by a deliberate decision to change mechanism rather than switch within class when evidence supports such a strategy. For HRR-positive disease, PARP-based therapy should be planned early, either as monotherapy after ARSI progression or as a PARP-ARSI combination depending on access, toxicity risk, and clinical urgency [26,27,28,29,30,31,32,33]. For PSMA-positive, taxane-naive disease after ARSI progression, PSMAfore supports RLT as an earlier mechanism switch compared with ARSI change, particularly when the goal is to avoid low-yield within-class switching while preserving performance status and marrow reserve [43,44]. For patients without actionable HRR findings and with PSMA-negative or heterogeneous disease, taxane chemotherapy remains central, and the key question becomes whether taxanes should be advanced earlier to avoid prolonged ineffective ARSI cycling. CARD supports cabazitaxel over ARSI switching in a critical post-docetaxel setting [23], and a taxane-sequencing framework that explicitly weighs competing mechanisms has been articulated [24].

Across these branches, toxicity management is part of sequencing rather than a parallel task. Anemia and cytopenias related to PARP combinations, marrow suppression from taxanes, and hematologic toxicity from RLT all draw from the same physiological reserve. The practical solution is to treat marrow reserve as a resource that must be preserved, monitored, and deliberately allocated. This principle has been emphasized by Marchetti et al., who contextualized PARP inhibitors alongside theranostic and radiometabolic approaches [36].

Implementation also depends on multidisciplinary coordination and local pathways. A multidisciplinary expert-panel recommendation provides practical guidance on coordinating urology, medical oncology, nuclear medicine, genetics, and supportive care to optimize delivery when ideal sequences are constrained by access or comorbidity [51]. This form of implementation guidance is particularly valuable for institutions establishing PSMA-targeted RLT programs or mainstreaming genetic testing.

## 7. Special Considerations

Special considerations often determine whether a sequence succeeds or fails. Cardiovascular disease, frailty, polypharmacy, and cognitive vulnerability influence ARSI selection and determine whether intensification can be sustained over years. A scoping review comparing outcomes after abiraterone versus enzalutamide in real-world populations highlights how toxicity profiles and outcomes may differ across patient subsets, reinforcing the importance of comorbidity-aware selection and monitoring plans when prolonged treatment durations are anticipated [52].

Genetic testing introduces additional implementation requirements. Germline results have implications for relatives, screening, and counseling capacity. The review provides counseling checklists and workflow recommendations that support equitable implementation and may reduce missed opportunities for cascade testing and risk-reduction strategies [3]. These considerations may be clinically relevant even when the immediate objective is treatment selection, because delays in counseling or uncertainty about result interpretation may slow actionable decisions.

Marrow reserve is the most important cross-cutting constraint in the ARSI-PARP-RLT era. PARP combinations frequently cause anemia and may exacerbate cytopenias [29,30,31,32,33], taxanes may suppress marrow function, and RLT requires careful monitoring of blood counts and renal function [42]. In patients with extensive bone metastases or baseline anemia, the sequencing plan should anticipate dose modifications and incorporate supportive care early. When marrow reserve is marginal, clinicians may reasonably choose regimens with a lower hematologic burden first, reserving therapies associated with clinically relevant hematologic toxicity for the period in which the anticipated benefit is greatest.

PSMA heterogeneity is a practical treatment-selection constraint rather than only an imaging issue. When PSMA expression is heterogeneous or clinically aggressive PSMA-low disease is suspected, clinicians should avoid relying exclusively on PSMA-targeted therapy and should consider mechanisms less dependent on target expression, including taxanes or clinical trials [4,42].

## 8. Ongoing Trials and Future Directions

Several ongoing and recently reported trials are testing whether PSMA-targeted RLT should move earlier in the prostate cancer treatment sequence. PSMAddition is testing RLT integration in metastatic hormone-sensitive prostate cancer, whereas UpFrontPSMA has explored earlier use of RLT in de novo high-volume metastatic hormone-sensitive disease [53,54].

In mCRPC, SPLASH and ECLIPSE indicate that the pre-taxane setting may become a broader PSMA-targeted RLT class space rather than a single-agent space, with treatment selection increasingly shaped by imaging eligibility, access, prior androgen receptor signaling inhibitor exposure, and marrow reserve [55,56,57,58]. Combination approaches, including RLT with immune checkpoint inhibition or PARP inhibition, are conceptually attractive but should be judged by whether efficacy gains can be achieved without compromising hematologic tolerance or subsequent treatment eligibility [59,60].

Beyond mCRPC, multiple trials are examining whether HRR vulnerability should be addressed in mCSPC rather than deferred until castration resistance. TALAPRO-3 evaluates talazoparib plus enzalutamide versus placebo plus enzalutamide in HRR-altered mCSPC [61]. AMPLITUDE tested niraparib added to abiraterone acetate plus prednisone in HRR-altered mCSPC and has been reported in a peer-reviewed randomized phase III publication, thereby strengthening the evidence base for earlier biomarker-enriched intensification [62]. These studies will clarify whether earlier integration of PARP inhibitors can delay castration resistance and improve long-term outcomes beyond those achieved with ARSI-based intensification alone.

Finally, alpha-emitting PSMA therapy is entering late-stage development to address the need for higher-linear-energy-transfer radiation and potentially deeper cytotoxicity in resistant or micrometastatic disease. Actinium-225 PSMA therapy is particularly attractive because alpha particles have a short tissue range and high linear energy transfer, but post-therapy imaging, lesion visualization, dosimetry, xerostomia, renal exposure, and marrow toxicity remain important challenges [63]. Lead-212 PSMA-targeted alpha therapy is also emerging, with first-in-human imaging experience supporting the feasibility of PSMA-directed 212Pb-based theranostics in mCRPC [64]. Late-stage trials of actinium-225 PSMA-617 are underway, including AcTFirst and PSMAcTION [65,66]. If these approaches are successful, the theranostic era will expand from a binary question of PSMA positivity to a more complex question of radionuclide class, absorbed dose, tumor heterogeneity, prior radioligand exposure, and sequencing relative to taxanes and PARP-based therapy.

## 9. Discussion: Controversies, Limitations, and Implementation Gaps

This review proposes an implementation-first sequencing framework, but several controversies should be emphasized. First, treatment intensification in mCSPC is now strongly supported, yet the optimal degree of intensification remains patient-dependent. Triplet therapy is most compelling for chemotherapy-fit men with aggressive de novo or high-volume disease, whereas the net benefit is less certain in frail patients, low-burden disease, and patients whose metastatic status has been reclassified by more sensitive PSMA PET imaging.

Second, mechanism switching in mCRPC is biologically and clinically rational, but it should not be interpreted as a rigid rule. Sequential AR pathway inhibition may still occur when access, toxicity, patient preference, or frailty limit alternatives. Nevertheless, AR-axis reactivation, AR splice variants, and preclinical cross-resistance provide a strong rationale for avoiding routine ARSI-to-ARSI substitution when taxanes, PARP-based therapy, PSMA-targeted RLT, or clinical trials are feasible [18,19].

Third, PARP–ARSI combinations illustrate both the promise and complexity of biomarker-guided intensification. The biological interaction between AR signaling and DNA repair supports co-inhibition [21,22], but clinical benefit is not uniform across HRR genes or across HRR-unselected populations. Therefore, implementation should incorporate gene-specific interpretation, anemia risk, marrow reserve, access, and patient goals rather than treating PARP–ARSI combinations as universally interchangeable first-line mCRPC options.

Fourth, PSMA-targeted RLT is changing the mCRPC sequencing landscape, but unresolved issues remain. PSMA PET is an essential selection tool, yet PSMA heterogeneity, discordant aggressive disease, renal function, marrow reserve, and local nuclear medicine capacity all influence deliverability. Dosimetry-guided approaches and personalized dosing may improve therapeutic optimization, but standardization and prospective validation are still needed before they can be considered routine requirements [50].

Finally, alpha-emitting PSMA therapies such as 225Ac-PSMA and 212Pb-PSMA are promising but remain investigational in many settings. Their future role will depend not only on response rates but also on salivary gland toxicity, renal exposure, marrow safety, post-177Lu sequencing, and practical access to radiopharmaceutical production and imaging workflows [63,64]. These uncertainties reinforce the central message of this review: sequencing is not a one-time choice but a longitudinal strategy that must integrate tumor biology, trial evidence, patient fitness, toxicity, and institutional infrastructure.

## 10. Conclusions

Clinical trials have redefined the management of metastatic prostate cancer, but the magnitude of clinical benefit depends on implementation and sequencing. In mCSPC, intensification with ARSIs and/or docetaxel improves survival compared with ADT alone, and triplet therapy provides additional benefit for selected chemotherapy-fit men with aggressive presentations. In mCRPC, switching within the ARSI class is often of limited value; randomized evidence supports changing mechanism, and the timing of taxane therapy remains a critical decision point in the context of expanding alternatives. HRR testing has evolved from optional to foundational, and care pathways that integrate counseling with actionable reporting enable timely PARP-based treatment decisions. PARP inhibitors and PARP-ARSI combinations have shifted first-line mCRPC toward rational combination strategies, while hematologic toxicity management and preservation of bone marrow reserve increasingly determine deliverability. In this sequencing context, PSMA PET functions chiefly as a gateway to PSMA-targeted RLT, which is now supported as a mechanism switch in selected patients with taxane-naive mCRPC and is being tested earlier in the disease continuum.

A clinician-facing sequencing mindset is therefore straightforward: intensify early when appropriate, test early and act on HRR findings, use PSMA imaging to open or close theranostic pathways, and change mechanism rather than merely change brand when the evidence indicates cross-resistance. The next generation of trials is evaluating whether these principles should be applied even earlier through first-line theranostics and biomarker-enriched intensification in mCSPC, and whether alpha emitters and combination regimens can provide more durable disease control for patients who progress on current standards.

## Figures and Tables

**Table 1:** Pivotal trials and practice-shaping evidence in mCSPC.

Trial	Key Population	Arms	Endpoints	Key Efficacy Outcome	Clinical Interpretation
CHAARTED [6]	mHSPC; disease-volume stratification prespecified; high-volume defined by visceral metastases and/or ≥4 bone lesions with ≥1 beyond the vertebral bodies or pelvis	ADT vs. ADT + docetaxel	OS	OS: 57.6 vs. 44.0 months; HR 0.61 (95% CI 0.47–0.80). Time to CRPC: 20.2 vs. 11.7 months; HR 0.61 (95% CI 0.51–0.72)	Supports upfront docetaxel, especially in chemotherapy-fit patients with high-volume or rapid-tempo disease
STAMPEDE (docetaxel arm) [7]	Men starting long-term hormone therapy for high-risk locally advanced, metastatic, or recurrent prostate cancer in a platform design	SOC vs. SOC + docetaxel	OS; FFS	OS: approximately 81 vs. 71 months; HR 0.78 (95% CI 0.66–0.93). Failure-free survival: approximately 37 vs. 20 months; HR 0.62 (95% CI 0.54–0.70)	Supports generalizability of early docetaxel, but interpretation for pure mCSPC should consider mixed eligibility
LATITUDE [8]	Newly diagnosed high-risk de novo mCSPC; high-risk defined by ≥2 of the following: Gleason score ≥8, ≥3 bone lesions, or visceral metastases	ADT vs. ADT + abiraterone + prednisone	rPFS; OS	Final OS: 53.3 vs. 36.5 months; HR 0.66 (95% CI 0.56–0.78). rPFS: 33.0 vs. 14.8 months; HR 0.47 (95% CI 0.39–0.55)	Strong chemotherapy-free intensification option for high-risk de novo disease; steroid and mineralocorticoid-related monitoring are important
ENZAMET [9]	Broad mHSPC population receiving testosterone suppression; early docetaxel allowed in a substantial subset	ADT ± enzalutamide (with SOC options)	OS	3-year OS: 80% vs. 72%; HR 0.67 (95% CI 0.52–0.86). Clinical PFS improved; HR approximately 0.40 (95% CI 0.33–0.49)	Demonstrates ARSI benefit in broad mHSPC; concurrent docetaxel and chronic toxicity should be considered
TITAN [10]	Broad mCSPC population including de novo and recurrent metastatic disease; prior docetaxel allowed in a minority	ADT ± apalutamide	rPFS; OS	24-month OS: 82.4% vs. 73.5%; HR 0.67 (95% CI 0.51–0.89). 24-month rPFS: 68.2% vs. 47.5%; HR 0.48 (95% CI 0.39–0.60)	Supports ARSI + ADT across risk and volume strata; rash, hypothyroidism, falls, fractures, and adherence require monitoring
ARASENS [11]	mHSPC planned for ADT + docetaxel; chemotherapy-fit population; most patients had de novo metastatic disease	ADT + docetaxel ± darolutamide	OS	OS: HR 0.68 (95% CI 0.57–0.80); 4-year OS 62.7% vs. 50.4%. Time to CRPC: HR 0.36 (95% CI 0.30–0.42)	Supports triplet therapy in selected chemotherapy-fit patients with aggressive disease; docetaxel fitness remains decisive
PEACE-1 [12]	De novo mCSPC in a 2 × 2 factorial design; docetaxel subgroup is most relevant to triplet therapy	ADT ± docetaxel ± abiraterone	rPFS; OS	In ADT + docetaxel population, OS: not reached vs. 4.43 years; HR 0.75 (95.1% CI 0.59–0.95). rPFS: 4.46 vs. 2.03 years; HR 0.50 (99.9% CI 0.34–0.71)	Supports abiraterone-based triplet therapy in de novo mCSPC; open-label factorial design and evolving SOC require careful interpretation
ARANOTE [16]	mHSPC treated without planned docetaxel; chemotherapy-free intensification setting	ADT ± darolutamide	rPFS (disease control)	rPFS: HR 0.54 (95% CI 0.41–0.71). OS immature at primary analysis	Expands chemotherapy-free ARSI options for patients in whom docetaxel is undesirable; mature OS data remain important

Abbreviations: ADT, androgen deprivation therapy; ARSI, androgen receptor signaling inhibitor; CI, confidence interval; CRPC, castration-resistant prostate cancer; FFS, failure-free survival; HR, hazard ratio; mCSPC, metastatic castration-sensitive prostate cancer; mHSPC, metastatic hormone-sensitive prostate cancer; OS, overall survival; rPFS, radiographic progression-free survival; SOC, standard of care; vs., versus.

**Table 2:** Pivotal trials and practice-shaping evidence in mCRPC.

Trial	Key Population	Arms	Endpoints	Key Efficacy Outcome	Clinical Implementation
CARD [23]	mCRPC previously treated with docetaxel and one AR-targeted agent; progression within 12 months on prior AR-targeted therapy	Cabazitaxel vs. alternate ARSI	rPFS; OS	OS: 13.6 vs. 11.0 months; HR 0.64 (95% CI 0.46–0.89). Imaging-based PFS: 8.0 vs. 3.7 months; HR 0.54 (95% CI 0.40–0.73)	Clinical anchor against reflex ARSI-to-ARSI switching after key exposures; supports mechanism switching when feasible
TOPARP-A [25]	Heavily pretreated mCRPC; DDR alterations evaluated as biomarkers of PARP sensitivity	Olaparib (single-arm)	Composite response; PFS; OS	Composite response: 33% overall; 88% in biomarker-positive vs. 6% in biomarker-negative patients. Median OS: 10.1 months overall	Proof of concept for PARP sensitivity in DDR-defective mCRPC; single-arm design limits comparative inference
PROfound [26,27]	HRR-altered mCRPC after prior ARSI; Cohort A included BRCA1/2 or ATM alterations	Olaparib vs. physician’s choice of enzalutamide or abiraterone	rPFS; OS	Cohort A rPFS: 7.4 vs. 3.6 months; HR 0.34 (95% CI 0.25–0.47). Final OS: 19.1 vs. 14.7 months; HR 0.69 (95% CI 0.50–0.97), despite crossover	Supports early HRR testing and PARP inhibitor use after ARSI progression; benefit is strongest and most actionable in BRCA1/2-altered disease
TRITON3 [28]	mCRPC with BRCA1/2 or ATM alteration after progression on one ARPI; no prior chemotherapy for mCRPC	Rucaparib vs. physician’s choice	rPFS	BRCA subgroup imaging-based PFS: 11.2 vs. 6.4 months; HR 0.50 (95% CI 0.36–0.69). ITT imaging-based PFS: 10.2 vs. 6.4 months; HR 0.61 (95% CI 0.47–0.80)	Confirms PARP inhibition as a core mechanism in selected HRR-altered mCRPC; ATM benefit is less clear than BRCA1/2
PROpel [29,30]	First-line mCRPC, all-comer population	Abiraterone + prednisone + placebo vs. abiraterone + prednisone + olaparib	rPFS; OS	rPFS: 24.8 vs. 16.6 months; HR 0.66 (95% CI 0.54–0.81). Final OS: 42.1 vs. 34.7 months; HR 0.81 (95% CI 0.67–1.00)	Supports PARP–ARSI intensification in first-line mCRPC, but patient selection, HRR status, anemia, and access remain critical
MAGNITUDE [31,32]	First-line mCRPC stratified by HRR status; HRR-negative cohort stopped for futility	Abiraterone + prednisone + placebo vs. abiraterone + prednisone + niraparib	rPFS; OS	HRR-positive rPFS: 16.5 vs. 13.7 months; HR 0.73 (95% CI 0.56–0.96). BRCA1/2 subgroup rPFS HR 0.53 (95% CI 0.36–0.79)	Supports biomarker-enriched PARP–ARSI use; HRR-negative futility is important for critical interpretation
TALAPRO-2 [33,34]	First-line mCRPC; all-comer and HRR-deficient cohorts analyzed	Enzalutamide + placebo vs. enzalutamide + talazoparib	rPFS; OS	Primary all-comer analysis: median rPFS not reached vs. 21.9 months; HR 0.63. Final OS: 45.8 vs. 37.0 months; HR 0.80 (95% CI 0.66–0.96)	Strong disease-control signal with OS support, but hematologic toxicity and gene-context interpretation remain central
VISION [40]	PSMA-positive mCRPC after prior ARPI and taxane chemotherapy; PSMA PET-selected	Standard care vs. standard care + 177Lu-PSMA-617	rPFS; OS	OS: 15.3 vs. 11.3 months; HR 0.62 (95% CI 0.52–0.74). Imaging-based PFS: 8.7 vs. 3.4 months; HR 0.40 (99.2% CI 0.29–0.57)	Established life-prolonging PSMA-RLT after ARPI and taxane exposure; PSMA selection and hematologic/renal monitoring are essential
TheraP [41,42]	PSMA-positive mCRPC after docetaxel; dual-tracer imaging used to exclude PSMA-low/FDG-discordant disease	Cabazitaxel vs. 177Lu-PSMA-617	PSA50 response; PFS; OS	PSA50 response: 66% vs. 37%. Mature follow-up showed broadly similar OS compared with cabazitaxel	Provides comparative evidence for PSMA-RLT in highly imaging-selected patients; strict selection criteria limit direct generalization
PSMAfore [43,44]	Taxane-naive PSMA-positive mCRPC after progression on one ARPI	Change of ARPI vs. 177Lu-PSMA-617	rPFS; OS	rPFS improved: approximately 12.0 vs. 5.6 months; HR approximately 0.43. Final OS was not statistically significant in the ITT population, with substantial crossover	Supports RLT as a mechanism switch over ARPI change in selected taxane-naive patients; OS interpretation and access require caution

Abbreviations: AR, androgen receptor; ARPI, androgen receptor pathway inhibitor; ARSI, androgen receptor signaling inhibitor; CI, confidence interval; DDR, DNA damage repair; FDG, fluorodeoxyglucose; HR, hazard ratio; HRR, homologous recombination repair; ITT, intention to treat; mCRPC, metastatic castration-resistant prostate cancer; OS, overall survival; PARP, poly(ADP-ribose) polymerase; PFS, progression-free survival; PSA50, prostate-specific antigen decline of ≥50%; PSMA, prostate-specific membrane antigen; rPFS, radiographic progression-free survival; RLT, radioligand therapy; vs., versus.

**Table 3:** Pragmatic sequencing matrix (“what to do next”) integrating HRR status, PSMA eligibility, fitness, and prior exposures.

Clinical Scenario	Key Assessment	Preferred Strategy	Avoid or Use Caution	Key Supporting Evidence
Newly diagnosed de novo high-volume mCSPC, chemotherapy-fit	Disease volume/risk, symptoms, ECOG PS, baseline laboratory data, comorbidities	Triplet therapy: ADT + docetaxel + ARSI	ADT alone unless clear contraindications to intensification	CHAARTED, ARASENS, PEACE-1 [6,11,12]
Newly diagnosed mCSPC, chemotherapy-unfit or frail	Frailty, falls/cognition, cardiovascular risk, steroid suitability, monitoring capacity	Chemotherapy-free intensification with ARSI + ADT	Docetaxel-based regimens when toxicity threatens deliverability	LATITUDE, ENZAMET, TITAN, ARANOTE [8,9,10,16]
Low-burden or PSMA PET-era oligometastatic mCSPC	Imaging context, local therapy candidacy, multidisciplinary review	ADT + ARSI; consider local or metastasis-directed therapy in selected cases	Chemotherapy overtreatment without clear expected benefit	EAU guideline; de-escalation/oligometastatic literature [1,4,14]
Transition to mCRPC after ARSI exposure, HRR pending	Confirm castration status, disease tempo, symptoms, tissue/ctDNA availability, PSMA PET if RLT is considered	Expedite HRR testing and select a mechanism-distinct option according to urgency	Prolonged routine ARSI-to-ARSI switching	CARD; AR-V7 and cross-resistance evidence [17,18,19,23]
HRR-positive mCRPC, especially BRCA1/2, taxane-naive	Gene context, germline/somatic status, hemoglobin, platelets, prior therapy	PARP-based therapy or PARP–ARSI combination according to approval, access, and toxicity risk	Treating all HRR alterations as biologically equivalent	PROfound, TRITON3, PROpel, MAGNITUDE, TALAPRO-2; AR–DNA repair biology [21,22,26,27,28,29,30,31,32,33]
PSMA-positive, taxane-naive mCRPC after one ARPI	PSMA PET eligibility; CBC and renal baseline; nuclear medicine access	PSMA-targeted RLT where approved and accessible	Default ARSI change when mechanism switch is feasible	PSMAfore, VISION, PSMA PET/RLT guidance [40,42,43,44,47]
PSMA-negative, PSMA-heterogeneous, or clinically discordant mCRPC	PSMA heterogeneity, disease tempo, symptoms, visceral disease, FDG discordance if available	Taxane chemotherapy or clinical trial	PSMA-targeted RLT without target suitability	TheraP selection framework; lineage plasticity literature [20,41,42,47]
Post-docetaxel and post-ARSI mCRPC	Prior taxane response, neuropathy, marrow reserve, PSMA PET if RLT is considered	Cabazitaxel; PSMA-RLT if PSMA-positive and accessible	Alternate ARSI switching as default	CARD, VISION, TheraP [23,40,41,42]
Extensive bone disease or baseline anemia in mCRPC	CBC trend, renal function, iron/B12/folate, transfusion need, prior marrow-toxic therapy	Choose the least hematologically compromising effective option compatible with disease urgency	Back-to-back therapies associated with clinically relevant hematologic toxicity without recovery/monitoring	PARP–ARSI trials; VISION; dosimetry literature [29,30,31,32,33,40,50]
Building an institutional sequencing pathway	HRR workflow, genetic counseling route, PSMA PET/RLT referral pathway, multidisciplinary coordination	Checklist-based pathway with early testing and early referral	Ad hoc late testing and late referral	Genetic-testing workflow, multidisciplinary recommendations, RLT guidance [3,42,51]

Abbreviations: ADT, androgen deprivation therapy; ARPI, androgen receptor pathway inhibitor; ARSI, androgen receptor signaling inhibitor; CBC, complete blood count; ctDNA, circulating tumor DNA; ECOG PS, Eastern Cooperative Oncology Group performance status; FDG, fluorodeoxyglucose; HRR, homologous recombination repair; mCRPC, metastatic castration-resistant prostate cancer; mCSPC, metastatic castration-sensitive prostate cancer; PARP, poly(ADP-ribose) polymerase; PET, positron emission tomography; PSMA, prostate-specific membrane antigen; RLT, radioligand therapy.

## Data Availability

No new data were created or analyzed in this study. Data sharing is not applicable to this article.

## Abbreviations

The following abbreviations are used in this manuscript:

ADT	Androgen deprivation therapy
ARSI	Androgen receptor signaling inhibitor
ctDNA	Circulating tumor DNA
DNA	Deoxyribonucleic acid
HRR	Homologous recombination repair
ISUP	International Society of Urological Pathology
mCRPC	Metastatic castration-resistant prostate cancer
mCSPC	Metastatic castration-sensitive prostate cancer
PARP	Poly(ADP-ribose) polymerase
PSMA	Prostate-specific membrane antigen
PSMA PET	Prostate-specific membrane antigen positron emission tomography
RLT	Radioligand therapy
